# Mitigation of Negative Effects of Chromium (VI) Toxicity in Faba Bean (*Vicia faba*) Plants through the Supplementation of Kinetin (KN) and Gibberellic Acid (GA3)

**DOI:** 10.3390/plants11233302

**Published:** 2022-11-29

**Authors:** Pravej Alam, Maged A. Azzam, Thamer Al Balawi, Vaseem Raja, Javaid Akhter Bhat, Parvaiz Ahmad

**Affiliations:** 1 Department of Biology, College of Science and Humanities, Prince Sattam Bin Abdulaziz University, Al-Kharj 11942, Saudi Arabia; 2 Department of Chemistry, College of Science and Humanities, Prince Sattam Bin Abdulaziz University, Al-Kharj 11942, Saudi Arabia; 3 University Centre for Research and Development Department, Chandigarh University Gharuan, Chandigarh 140413, India; 4 International Genome Center, Jiangsu University, Zhenjiang 212013, China; 5 Department of Botany, GDC Pulwama, Pulwama 192301, India

**Keywords:** antioxidants, chromium, faba bean, gibberellic acid, kinetin, antioxidant system

## Abstract

The present study was carried out to explore the possible role of kinetin and gibberellic acid (GA3) on faba bean under chromium (Cr) stress. Cr treatment negatively affected growth and biomass production, reduced photosynthetic pigments, and inhibited photosynthesis, gas exchange parameters, antioxidant enzymes, and the glyoxylase cycle. Moreover, Cr stress enhanced the production of malondialdehyde (MDA, 216.11%) and hydrogen peroxide (H_2_O_2_, 230.16%), electrolyte leakage (EL, 293.30%), and the accumulation of proline and glycine betaine. Exogenous application of kinetin and GA3 increased growth and biomass, improved pigment contents and photosynthesis, as well as up-regulated the antioxidant system by improving the antioxidant enzyme activities and the content of nonenzymatic components, and the glyoxylase cycle. Additionally, kinetin and GA3 application displayed a considerable enhancement in proline (602.61%) and glycine betaine (423.72), which help the plants to maintain water balance under stress. Furthermore, a decline in Cr uptake was also observed due to kinetin and GA3 application. Exogenous application of kinetin and GA3 ameliorated the toxic effects of Cr in faba bean plants, up-shooting the tolerance mechanisms, including osmolyte metabolism and the antioxidant system.

## 1. Introduction

The past few decades have witnessed an alarming increase in heavy metals because of rapid industrialization and urbanization. These heavy metals are often categorized as soil contaminants; unrestrained disposal intensifies their concentration, which encourages plant stress [1]. To combat the heavy metal stress, plants thriving under such contaminated environments often display several morphological, physiological, and biochemical alterations [2]. Among several naturally occurring heavy metals, chromium (Cr) is potentially hazardous to both flora and fauna [3]. Chromium is considered an important pollutant, and many sources contribute this pollutant to the environment such as metal smelters, leather tanning, steel industries, fertilizer and pesticide use, as well as emissions from industries [4,5]. In soil, Cr mainly exists in two ionic forms, Cr^3+^ (III) and Cr^6+^, and both are toxic to plants. However, the higher stability and reactive nature of Cr^6+^ are the important factors that make this form of Cr more toxic compared to other forms existing in the soil by negatively affecting plant growth and development [4,6]. The availability of Cr in plants is facilitated via the complexes formed by Cr with organic compounds in soil; however, its accumulation and translocation depend upon its oxidation state [7]. Under Cr stress, several physiological, biochemical, and metabolic impairments have been reported in plants. Major toxicity symptoms in plants resulting from Cr stress include stunted growth and chlorosis [8]. Chromium stress impedes numerous substantial plant activities including physiological aspects, membrane integrity, metabolic activities, and biochemical processes, such as inhibition of the electron transport chain and photosynthesis in the mitochondrion and chloroplast [8,9,10]. In addition, Cr toxicity severely inhibits the availability of mineral nutrients that, in turn, negatively inhabits plant growth and development [5]. Moreover, in plants, Cr stress has been reported to cause oxidative stress, leading to the over-production of reactive oxygen species (ROS) that causes damage to the cells and even to cell death [11]. However, to overcome metal stress leading to oxidative damage, plants have developed several intricate mechanisms by enhancing the efficient antioxidant system and production of osmolytes such as proline and glycine betaine [12,13].

Many recent studies have documented that phytohormones are versatile molecules that are helpful in combating different environmental stresses [9,14,15]. In order to overcome Cr-stress-mediated phytotoxicity, it is imperative to adopt eco-friendly techniques. Polyamines, amino acids, and phytohormones are some eco-friendly physiochemical approaches that maintain soil health [4,8]. For example, the cytokinins showed a role in overcoming abiotic stress by improving growth [16], regulating the cell cycle [17], homeostasis of the phytohormones and gene expression [15], as well as improving photosynthesis [18]. Recently, kinetin has been recognized as a key player among cytokinins for its role in the mitigation of oxidative stress and promotes growth [18,19]. Cytokinins promote cell division, and improve chlorophyll synthesis and organ development [20].

Gibberellins (Gas), a large family of diterpenoid tetracyclic compounds, comprise an assemblage of plant growth regulators associated with seed germination, plant growth, dormancy breaking, improved water uptake, and signaling [21,22]. Several studies have demonstrated that gibberellins are promising biological molecules that play a pivotal role in alleviating different types of abiotic and biotic stresses [23,24,25]. In addition to acting as signaling molecules, gibberellins enhance developmental processes by counteracting stressful conditions, uplifting growth, and enhancing plant immunity [26,27]. Additionally, diminutive evidence is accessible from the literature that highlights the role of plant growth played by GAs under stress conditions that make GAs the prime targets of the present research.

Among different legume crops grown in the world, the faba bean (*Vicia faba* L.) is one of the oldest crops and ranks sixth in their production [28]. This legume crop is also known by different common names such as field beans, broad beans, and horse beans [28]. In developing countries, beans are vital components of human nutrition as it contains a sufficient protein diet for humans [29]. Even at lower metal concentrations, a severe clampdown of biomass production in leguminous crops has been reported [30,31]. With regard to metal tolerance and accumulation in faba bean under Cr stress, however, little information is available. Phytohormones have emerged as the hub of modern research studies; however, from the literature, there is no evidence that highlights the role of kinetin and GA3 in Cr stress mitigation. In light of the earlier cited conceivable validation, the present investigation was commenced to describe the role and to unfold the possible mechanism of kinetin and GA3 in Cr stress tolerance in the faba bean under individual and/or combined application of kinetin and GA3.

## 2. Results

### 2.1. Growth and Biomass Yield

**Figure 1:**
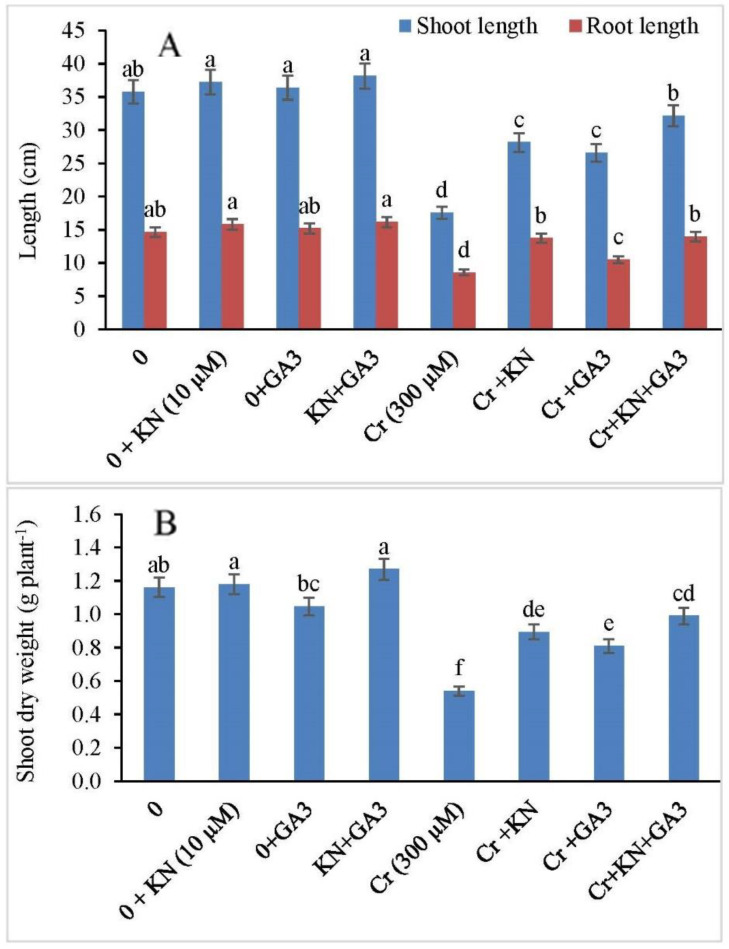
Effect of kinetin (KN) and gibberellic acid (GA3) on (**A**) shoot and root length, and (**B**) shoot dry weight in faba bean under Cr toxicity. The letters (a–f) denote significant difference at *p* < 0.05. Data represent mean ± SE (n = 5).

In the present study, we observed no significant difference in faba bean plants treated individually either with kinetin or GA3. However, exposure to 300 µM of Cr stress revealed a significant reduction in the shoot length by 50.93%. Additionally, when the Cr-treated faba bean plants were subjected to kinetin priming, the shoot length showed an increase of about 21.30%. Similarly, GA3 application to Cr-stressed plants also resulted in increased shoot length (25.77%). However, combined treatments of kinetin and GA3 to Cr-stressed plants showed a dramatic increase in the shoot length by 83.19% (Figure 1A).

In the case of root length, no significant change was recorded in faba bean plants treated either with kinetin or GA individually; however, the Cr stress (300 µM) negatively affected the root length (Figure 1A), and a significant decrease in root length was observed as 41.63%. Under Cr stress, the application of kinetin (10 µM) and GA3 individually improved the root length by 60.46% and 22.33%, respectively, in faba bean plants. However, the combined application of kinetin and GA3 significantly improved the root length in faba bean plants under Cr stress. The combined application increased the root length by 63.15% in comparison to the control.

Faba bean plants treated with kinetin or GA3 without Cr stress showed almost the same dry weight with respect to the control (Figure 1B). However, Cr exposure decreased the dry weight in faba bean plants by 53.44%. Exposure of Cr-stressed plants to kinetin and GA3 alone or in combination improved the dry weight per plant. Kinetin and GA3 individually improved dry matter by 64.81 and 50.00%, respectively, while the combined application of kinetin and GA3 showed a much higher increase (83.33%) in dry weight than the individual application of these hormones.

### 2.2. Chromium Accumulation and Translocation Factor

In the plants subjected to Cr stress, higher levels of Cr accumulation of 57.48 mg kg^−1^ DW were observed in the root tissue followed by 31.43 mg kg^−1^ DW in the shoots in faba bean plants (Table 1). Supplementation of kinetin to the Cr-stressed plants decreased the Cr accumulation to 19.25 mg kg^−1^ DW and 42.15 mg kg^−1^ DW in the shoot and root, respectively. The combined application of kinetin + GA3 to Cr-stressed plants reduced Cr accumulation to 15.48 mg kg^−1^ DW and 32.72 mg kg^−1^ DW in the shoot and root, respectively.

The translocation factor showed a declined trend under the individual and combined application of kinetin and GA3 (Table 1). Kinetin application alone decreased the TF in faba bean plants by 11.48%, whereas GA3 application reduced the TF up to 8.97%. However, combined supplementation of kinetin and GA3 showed a greater decrease of 13.36% in TF.

### 2.3. Pigment Systems, Photosynthesis, and Gas Exchange Parameters

**Figure 2:**
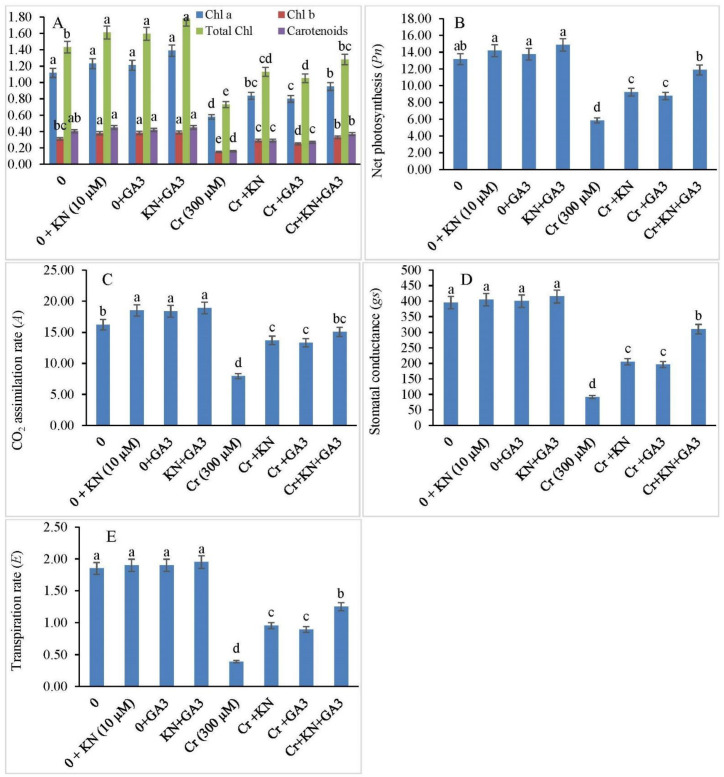
Kinetin (KN) and gibberellic acid (GA3) enhance (**A**) chlorophyll and carotenoid content (mg g^−1^ FW), (**B**) net photosynthesis, *Pn* (µmol m^−2^ S^−1^), (**C**) CO_2_ assimilation rate *A*, (µmol (CO_2_) m^−2^ S^−1^), (**D**) stomatal conductance (mmol m^−2^ S^−1^), and (**E**) transpiration rate (mmol H_2_O m^−2^ S^−1^) in faba bean under Cr toxicity. The letters (a–e) denote significant difference at *p* < 0.05. Data represent mean ± SE (n = 5).

Pigment contents, viz., Chl a, Chl b, TChl, and carotenoids, showed no significant difference among the kinetin or GA3-treated as well as control faba bean plants (Figure 2A). However, the Cr stress was found to decrease pigment content significantly in faba bean plants. In comparison to the control, we observed a reduction of 48.21%, 51.61%, 48.95%, and 60.00% in Chl a, Chl b, total Chl, and carotenoid content, respectively, under Cr stress in faba bean plants. Kinetin application enhanced Chl a, Chl b, total Chl, and carotenoid content by 25.00%, 6.45%, 20.97%, and 27.50%, respectively, in Cr-stressed plants. Similarly, the GA3 exposure under Cr stress also showed an augmentation in pigment content. For example, the GA3 application increased Chl a, Chl b, total Chl, and carotenoid content by 28.57%, 19.35%, 26.57%, and 32.50%, respectively, in Cr-treated plants relative to nontreated GA3 plants. Compared to individual effects of kinetin and GA3, combined treatment of kinetin and GA3 had a prominent effect on pigment enrichment (both chlorophyll and carotenoid). Combined treatment increased Chl a, Chl b, total Chl, and carotenoid content by 15.17%, 6.06%, 10.48%, and 7.5% in faba bean plants under Cr stress, respectively.

In this study, we observed no significant statistical difference in net photosynthetic rate (*Pn*), CO_2_ assimilation rate (*A*), stomatal conductance (*gs*), and transpiration rate (*E*) among the kinetin or GA3-treated and control faba bean plants (Figure 2B–E). However, all the attributes related to photosynthesis were severely affected under Cr stress. As compared to the control, the faba bean plants exposed to Cr displayed a decrease of about 55.47%, 50.98%, 76.76%, and 48.64% in *Pn*, *A*, *gs*, and *E*, respectively. Moreover, an increase in all the photosynthetic attributes was observed when Cr-treated plants were exposed to kinetin and GA3 alone or in combination. Combined kinetin and GA3 application demonstrated a considerably higher photosynthetic rate (102.73%), CO_2_ assimilation rate (100.70%), stomatal conductance (237.80%), and transpiration (220.51%) in comparison to control plants.

**Figure 3:**
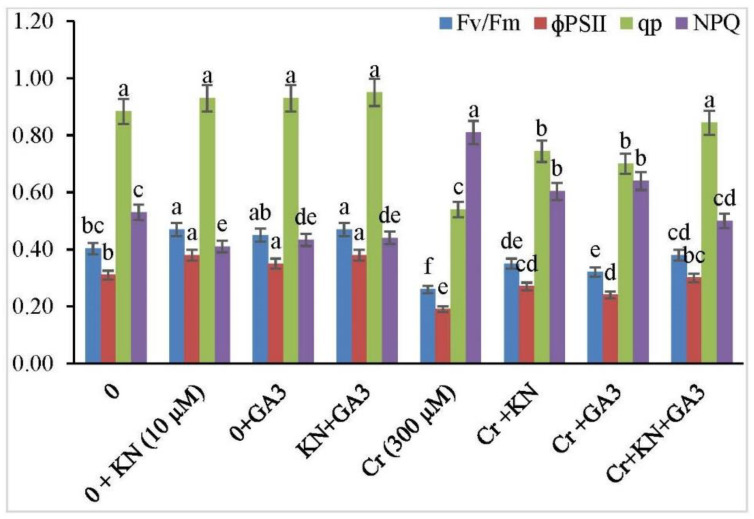
Application of kinetin (KN) and gibberellic acid (GA3) regulates chlorophyll fluorescence in faba bean under Cr toxicity. The letters (a–f) denote significant difference at *p* < 0.05. Data represent mean ± SE (n = 5).

In the absence of Cr treatment, no significant statistical difference was observed in chlorophyll florescence parameters (Figure 3) between faba bean plants not treated with kinetin or GA3, and those supplemented with either kinetin or GA3. Nonetheless, all the attributes related to chlorophyll florescence were significantly reduced under Cr stress. It is pertinent to mention that Cr-treated plants supplemented with kinetin or GA3 alone or in combination (kinetin and GA3) suffered less damage as compared to plants treated with Cr alone. Cr treatment alone decreased the PSII efficiency (Fv/Fm), photochemical efficiency (qp), and quantum yield of PSII (ΦPSII) by 35.00%, 38.63%, and 38.70%, respectively. However, exposure of Cr-treated plants to kinetin and GA3 individually improved all the chlorophyll-florescence-related parameters. Moreover, an increase in the chlorophyll-florescence-related parameters was observed when Cr-treated plants were exposed to the combined application of kinetin and GA3. Additionally, an increase in nonphotochemical quenching (NPQ) was observed in faba bean plants under Cr stress as compared to control plants, and an increase of 52.83% in NPQ was observed under Cr stress. However, the individual application of kinetin and GA3 decreased the NPQ by 25.92% and 20.98%, respectively. The combined application of kinetin and GA3 brought about a greater decrease (38.27%) in the level of NPQ in Cr-treated plants.

### 2.4. Relative Water Content, and Proline and GB Content

**Figure 4:**
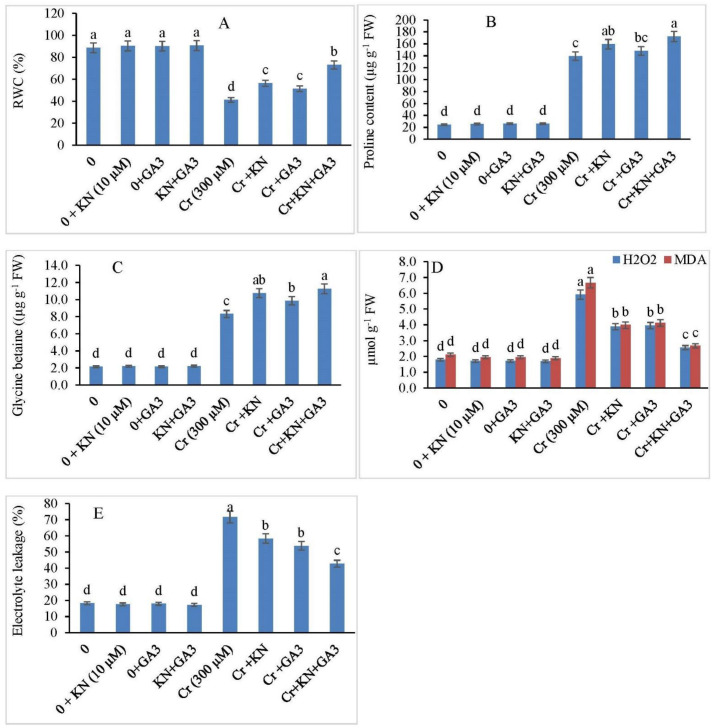
Effect of kinetin (KN) and gibberellic acid (GA3) on (**A**) RWC, (**B**) proline content, (**C**) glycine betaine content, and (**D**) oxidative stress biomarkers (H_2_O_2_ and MDA) and (**E**) EL in faba bean under Cr toxicity. The letters (a–d) denote significant difference at *p* < 0.05. Data represent mean ± SE (n = 5).

No significant difference in RWC was observed between the control, as well as kinetin and GA3-treated plants; however, the Cr stress decreased the RWC significantly by 53.44% relative to control plants (Figure 4A). Furthermore, a remarkable increase in RWC was observed in Cr-stressed plants upon treatment with kinetin and GA3. Kinetin alone enhanced the RWC by 36.54%, while GA3 increased the RWC up to 42.12% with respect to Cr-alone-treated plants. Additionally, the kinetin and GA3 in combination proved to be more operational in stress alleviation by increasing the RWC by 76.93% over Cr-alone-treated plants.

In comparison to the control, plants treated with kinetin and GA3 exhibited no significant difference in proline and GB accumulation when compared with the control (Figure 4B,C). Plants under Cr stress displayed a notable enhancement in proline and GB accumulation. With respect to the control, under Cr stress, the proline content and GB accumulation increased by 467.81% and 286.51%, respectively, in faba bean plants. However, the individual application of kinetin and GA3 further improved proline and GB accumulation under stress conditions. Kinetin application increased the proline content and GB accumulation by 549.50% and 400.00%, respectively. Similarly, the GA3 application increased the proline and GB content by 504.57% and 358.13%, respectively. Furthermore, the combined (kinetin and GA3) application under Cr stress proved to be very effective in proline and GB accumulation. Combined hormone application increased the proline and GB content by 602.61 and 423.72%, respectively.

### 2.5. H_2_O_2_, MDA Content, and EL

With respect to the control, plants treated with kinetin and GA3 individually displayed no statistical difference in H_2_O_2_, MDA, and EL (Figure 4D,E). However, plants exposed to Cr stress displayed augmented levels of H_2_O_2_, MDA, and EL. In comparison to the control, the Cr stress alone increased H_2_O_2_, MDA, and EL by 230.16%, 216.11%, and 293.30%, respectively. Furthermore, kinetin application under Cr stress decreased H_2_O_2_, MDA, and EL by 116.75%, 89.09%, and 220.03%, respectively, over the control plants. Similarly, the individual application of GA3 decreased H_2_O_2_, MDA, and EL by 120.67%, 95.26%, and 195.11%, respectively. Additionally, declines of about 56.68%, 26.54%, and 134.46% were observed in H_2_O_2_, MDA, and EL, respectively, in faba bean plants under combined application of kinetin and GA3.

### 2.6. Antioxidants Enzyme Activity

**Figure 5:**
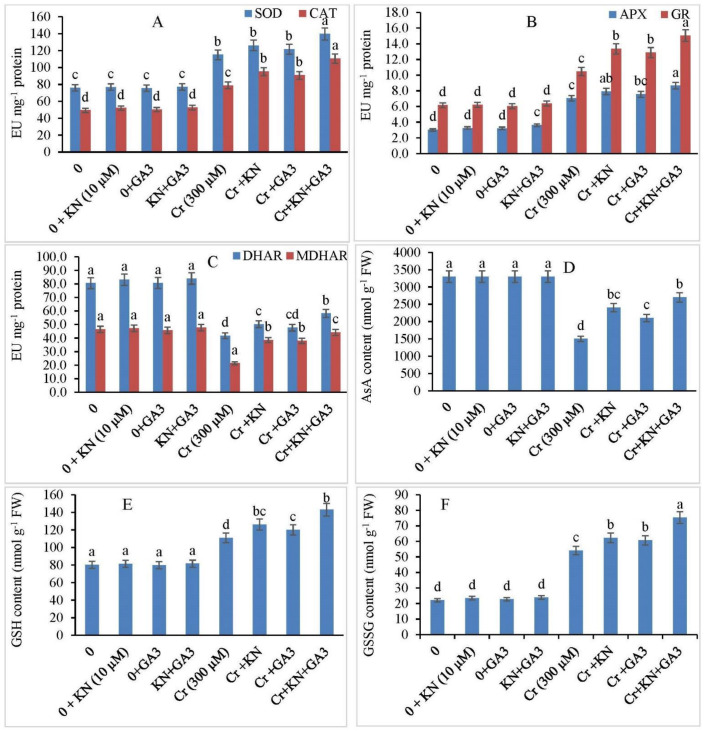
Supplementation of kinetin (KN) and gibberellic acid (GA3) regulates the activity of (**A**) SOD and CAT, (**B**) APX and GR, (**C**) DHAR and MDHAR, (**D**) AsA content, (**E**) GSH content, and (**F**) GSSG content in faba bean under Cr toxicity. The letters (a–d) denote significant difference at *p* < 0.05. Data represent mean ± SE (n = 5).

To explore the possible functional role of kinetin and GA3 on antioxidant enzymes in faba bean plants under Cr stress, the plants were treated with kinetin and GA3 individually or in combination. These results concomitant with the activities of the antioxidant enzymes are illustrated in Figure 5A–C. Compared to the control, plants treated with either Kinetin or GA3 displayed no significant statistical difference in antioxidant enzyme activities of faba bean plants. However, when the plants were exposed to Cr stress, a significant increase was observed in the activities of several antioxidant enzymes. Cr stress alone increased the activities of SOD, CAT, APX, and GR by 51.34%, 59.77%, 134.21%, and 70.24, respectively, relative to those of control plants. Kinetin application under Cr stress further increased the activity of SOD (65.78%), CAT (92.72%), APX (162.79%), and GR (117.07%) relative to control plants. Similarly, the GA3 application enhanced the activity of SOD, CAT, APX, and GR by 59.21%, 83.85%, 150.83%, and 109.43%, respectively, compared to control plants. Additionally, the combined application of kinetin and GA3 further enhanced the antioxidant enzyme activities of SOD, CAT, APX, and GR by 82.89%, 122.89%, 187.37%, and 144.87%, respectively, relative to control plants (Figure 5A,B).

Antioxidant activities prompted us to measure the activities of DHAR and MDHAR, and we observed a sharp decline in the activity of DHAR and MDHAR by 48.15% and 53.85%, respectively, in faba bean plants under Cr stress in comparison to control plants (Figure 5C). However, when Cr-stressed plants were treated with kinetin, an increase of about 37.65% and 16.82% was observed in the activities of DHAR and MDHAR, respectively. Similarly, the GA3 application also showed an improvement in DHAR and MDHAR activity by 48.89% and 18.01%, respectively, in comparison to Cr-stressed plants. Furthermore, combined kinetin and GA3 application remarkably improved the DHAR and MDHAR activity under Cr stress. Under the combined application of kinetin and GA3, the DHAR increased by 27.83% and MDHAR increased by 4.81% in comparison to Cr-treated plants alone. The results demonstrate that kinetin and GA3 either individually or in combination helped the faba bean plants to maintain the DHAR and MDHAR pool under Cr toxicity.

### 2.7. Kinetin and GA3 Application Maintains Ascorbic Acid (AsA), GSH, and GSSG Ratio

No significant statistical difference was observed in AsA, GSH, and GSSG content in control plants, and the ones treated with kinetin and GA3 (Figure 5D–F). However, under Cr stress, plants showed a sharp decline of about 54.54% in AsA levels. Moreover, we observed an improvement in the AsA levels in Cr-treated plants when supplemented with kinetin and GA3 either alone or in combination. The combined kinetin + GA3 treatment increased AsA levels up to 40.00% in plants subjected to Cr stress relative to those of the plants treated with Cr alone.

During the study, an increase of about 38.43% in GSH concentration was observed in faba bean plants under Cr stress as compared to nontreated plants; however, the application of kinetin and GA3 to Cr-stressed pants further increased the GSH content by 13.51% and 15.15%, respectively, in comparison to Cr-alone-treated plants. Additionally, the Cr-treated plants supplemented with combined kinetin + GA3 further enhanced the GSH activity by 78.34% with respect to Cr-alone-treated plants. The Cr stress enhanced the GSSG content by 144.77% relative to control plants, However, further enhancements by 181.86% and 174.58% were observed by the supplementation of kinetin and GA3 individually, respectively, to the Cr-treated plants in comparison to Cr-alone-treated plants. The combined application of kinetin + GA3 to Cr-stressed plants further enhanced the GSSG content by 240.66% with respect to Cr-alone-treated plants.

### 2.8. Methylglyoxal (MG) and Glyoxalase System

**Figure 6:**
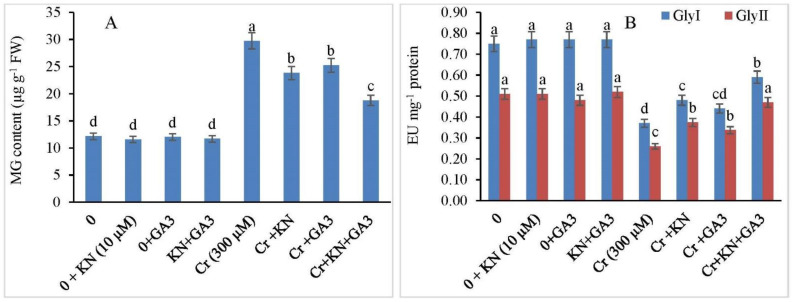
Application of kinetin (KN) and gibberellic acid (GA3) regulates (**A**) MG content (µmol g^−1^ FW) and (**B**) GlyI and GlyII (µmol min^−1^ mg^−1^ protein) in faba bean under Cr toxicity. The letters (a–d) denote significant difference at *p* < 0.05. Data represent mean ± SE (n = 5).

Chromium stress in faba bean plants enhanced MG levels up to 145.05% as compared to control plants (Figure 6A). However, the Cr-treated faba bean seedling when supplemented with kinetin and GA3 individually showed a significant reduction by 96.12% and 36.00%, respectively, as compared to Cr-alone-treated plants. Cr-stressed plants showed a maximum decrease of about 54.53% in MG levels when kinetin and GA3 were applied in combination with respect to Cr-alone-treated plants.

Under Cr stress, reduced GlyI and GlyII activities were observed in faba bean plants (Figure 6B). GlyI and GlyII activities declined by 50.66% and 49.01% respectively, under Cr stress in comparison to nontreated plants. Relative to Cr-alone-treated plants, kinetin enhanced the activities of both GlyI (36.00%) and GlyII (27.45%) enzymes in Cr-stressed plants. Foliar application of GA3 to Cr-stressed plants also enhanced the GlyI and GlyII by 41.33% and 33.33% respectively, over Cr-alone-treated plants. Higher activities of 21.33% in GlyI and 7.84% in GlyII were observed when kinetin + GA3 was applied in combination to cadmium-stressed plants as compared to Cr stress alone.

## 3. Discussion

### 3.1. Plant Growth and Biomass

Plants being nonmobile in nature are often subjected to environmental stresses including heavy metals [32]. Among the heavy metals, Cr has been reported to adversely affect plant growth and development by inhibiting several metabolic processes [33,34]. In the present study, a significant reduction in growth (shoot and root length) and biomass production was observed in the faba bean plants under Cr stress. In agreement with our results, several recent studies have also documented a decline in growth and biomass in different plants such as *Brassicca napus*, wheat, and Arabidopsis under Cr stress [35,36,37]. The reduction in plant growth by Cr phyto-toxicity has been suggested to result from the degradation of chlorophyll pigments [38,39], disturbed nutrient uptake balance [33], ROS overproduction, destruction of cellular ultrastructure [40], and disorganization in antioxidant defense machinery [33]. Hence, it is a prerequisite to improve the Cr stress tolerance in faba bean to avoid its negative effect on plant growth. In this regard, we used two plant hormones, viz., kinetin and GA3, to explore their role in the Cr tolerance in faba bean plants. Our results demonstrated that kinetin and GA3 application individually as well as in combination considerably improved growth and biomass in faba bean under Cr stress. The application of GA3 has been previously documented to significantly improve plant growth and biomass in leaf lettuce [41] and *Vigna radiata* [23]. The GA3-mediated promotion of growth and biomass under stress can be ascribed to the promoted synthesis of DNA, RNA, nuclear material, and proteins [42,43], improved enzyme activity, and optimal nutrient uptake [23,44]. Moreover, improved growth performance was observed in kinetin-treated faba bean plants exposed to Cr stress. Several researchers have demonstrated the ameliorative effect role of kinetin under different abiotic stress conditions [18,19,45]. Kinetin has been suggested to improve growth and biomass in faba bean plants under stress conditions by enhancing the uptake of nutrients [46], hormones [47], as well as the accumulation of polyamines. Our results demonstrated that the combined application of kinetin and GA3 has a more pronounced effect on growth and biomass yield in the faba bean plants under Cr stress. The synergetic action of kinetin and GA3 on plant growth and biomass under various abiotic stress conditions has also been documented in chickpea [48], barley, wheat, cucumber, alfalfa, tomato, lettuce [49,50], and Chrysanthemum [51].

Heavy metal toxicity in plants depends on the amount, distribution, and partitioning of metal in different organs [52,53]. Remarkably, roots are the first plant organs that come in direct contact with metal ions, and, thus, roots play an important role in the regulation of metal uptake [54,55]. In the present investigation, Cr exposure considerably increased the accumulation and uptake of Cr, in both the roots and leaves of faba bean plants. Our results are in agreement with the findings previously observed in barley, *Salvinia* [56], and wheat [57]. We observed a higher Cr accumulation in roots compared to the leaves. However, kinetin and GA3 application alone as well as in combination considerably decreased the Cr translocation and uptake from roots to leaves. Kinetin and GA3 caused a reduction in Cr translocation, and uptake might be related to the defensive role of these hormones in membrane stability [58], which, in turn, prevented Cr from entering into the cytoplasm. Hence, the increase in the phytohormone-mediated increment in plant growth and biomass has been suggested to likely result from low metal translocation under Cr stress [58].

### 3.2. Photosynthetic Efficiency

The extent of damage to the plant’s photosynthetic machinery due to various environmental perturbations can be best explained through their primary effect on the disruption of the photosynthetic pigments [5,23]. Our study documented the significant reduction in the chlorophyll and carotenoid pigments under Cr stress, similar to those previously reported by different authors in various plant species [33,59,60]. It has been suggested that the reduction in the chlorophyll and carotenoid pigments due to heavy metal stress including Cr might have resulted from the inhibition of enzymatic machinery involved in the pigment biosynthesis and photosystem damage [33,61,62]. In our study, the application of kinetin and GA3 individually or in combination increased the photosynthetic pigments under Cr stress, but the combined effect of kinetin and GA3 was more profound, similar to that previously demonstrated by Terzi and Kocaçalişkan [50]. Although there is no direct evidence available demonstrating the role of GA3 on the photosynthetic pigments under Cr stress in plants, GA3 application has been reported to positively affect the biosynthesis of photosynthetic pigments for the case of other heavy metal stresses such as *Chlorella vulgaris* [63] and *Vigna radiata* [23]. These authors suggested that increased pigment content under metal stress upon foliar application of GA3 might have been due to reduced oxidative damage resulting from GA3-enhanced antioxidant defense mechanisms. In agreement with our findings, the kinetin application to the Cr-stressed plants has been documented to enhance pigment content [17,64]. Therefore, the restoration in photosynthetic pigments under kinetin and GA3 supplementation suggested their important role in protecting the photosynthetic apparatus against Cr-mediated photodynamic damage as reported in previous studies [15,64].

The reduction in the photosynthetic gas exchange parameters, viz., *Pn*, *A*, *E*, and *gs*, under Cr toxicity is in accordance with those observed in barley [60], rice [65], and cauliflower [33]. In addition, many other recent studies have also reported severe reductions in gas exchange parameters under Cr toxicity [10,66]. Many authors have suggested that the Cr-induced reduction in the leaf gas exchange parameters may be attributed to altered enzyme activities, which leads to protein dysfunction [10,65,66]. Similar to our results, the GA3 application has also been previously reported to enhance the gas exchange parameters in different plant species such as jute [67] and mungbean [23] under heavy metal stress. It has been reported that GA3 enhanced the photosynthetic activity by increasing the CO_2_ assimilation rate [68], Rubisco enzyme activity [69], and nutrient uptake [23], which, in turn, increased the plant growth and development. In addition, several researchers have reported an enhancement in photosynthesis and gas exchange parameters by kinetin application under heavy metal stress in different plants [64,70,71], similar to that reported in the present study. The enhanced gas exchange under Cr stress by kinetin application might be attributed to the higher antioxidant activity that can prevent ROS-induced photosynthetic damage [64]. The combined effect of kinetin and GA3 on these gas exchange parameters was found to be more prominent combined with their individual effect, similar to that reported by Terzi and Kocaçalişkan [50].

Under severe environmental perturbations, chlorophyll fluorescence is extensively implied to enumerate stress tolerance and acclimation in plants [72]. During the present study, a significant decrease was observed in the Fv/Fm, ΦPSII, and qP under Cr stress, and these results are consistent with those reported in Wheat [73], Tomato, and *Brassica napas* [39]. Impaired PSII electron flow under Cr stress can cause photo-inhibition that enhances the obliteration in antenna molecules [74,75]. Electron transport from the primary to secondary acceptor is blocked at the PSII acceptor side that drastically reduces the ratio Fv/Fm [39,76]. Negative effects on various photosynthetic parameters such as ΦPSII, qP, Fv/Fm ratio, and NPQ were reversed when Cr-stressed faba bean plants were treated with GA3 and kinetin alone or in combination. GA3 application improved chlorophyll-fluorescence-related parameters under heavy metal stress and has been reported by several authors [24,67]. Under Cr stress, GA3 allows the plant to effectively uphold their electron pool, which results in the reduction in PSII photoinhibition as well as the maintenance of the ΦPSII, Fv/Fm ratio, and qp. Similar findings have been reported in eggplant [77], *Mentha piperita* [78], and cucumber [79]. During the present study, a decline in NPQ was observed in faba bean seedlings under Cr stress conditions, and the results are consistent with observations in *Brassica juncea*, cauliflower [33], and *Halienthus annus* [66], which also documented a decreased trend in various photosynthetic attributes under Cr stress. The findings advocate that GA3 helps in maintaining the integrity of thylakoid membranes, and at the same time, prevents PSII from over-excitation.

### 3.3. Relative Water Content, and Proline and GB Contents

RWC is considered an important indicator for stress [80,81]. Chromium toxicity was observed to decrease the RWC in faba bean plants during the present investigation. In accordance with our study, several studies have also reported significant decreases in RWC under Cr toxicity in different plant species such as *Phyllanthus emblica*, tomato, and maize [80,82]. These authors suggested that the decrease in the RWC under Cr stress might be attributed to the decrease in the root surface area [80,83], which, in turn, has decreased the root absorption and assimilation rate of Cr in the roots, in order to protect the aerial parts from high Cr accumulation by satisfying optimal water uptake to the aerial parts [80,81]. The application of kinetin and GA3 individually or in combination increases the RWC under Cr stress, but the combined use of kinetin and GA3 has a more profound effect on the RWC. A similar observation was earlier reported under heavy metal stress in *Vigna angularis* and mungbean [23]. Foliar application of GA3 alleviated the severe effects of Cr by augmenting RWC levels through the reduction in metal accumulation [23], increased root elongation and shoot biomass, and enhanced enzyme activities [84].

Proline and GB act as osmoregulators and protect the cells from osmotic stress [85]. Proline is a multifunctional molecule that acts as a molecular chaperone, enhances enzyme activity, prevents membrane damage, and scavenges the ROS [86,87,88,89]. Furthermore, under stressful environments, GB protects the photosynthetic apparatus, enhances the enzyme activity, helps in osmotic adjustments, and also prevents protein damage [90,91,92]. Proline and GB have been reported as strong osmolytes, and their accumulation was reported under Cr stress [93,94]. Cr stress enhanced the proline and GB content in different plants and has also been investigated in different crops such as *Sorghum bicolor* [55], Chenopodium quinoa [95], *Brassica juncea* [96], and *Sorghum bicolor* [97]. However, the GA3 supplementation alone further augmented the proline content and GB under Cr stress in the present study, and our results corroborate those observed by earlier works in different crops of *Solanum lycopersicum*, *Helianthus annuus*, and *Capsicum annum* [4,98]. GA3-enhanced proline accumulation might be due to the enhanced activity of enzymes related to the synthesis of proline or reduction in catabolizing enzymes [23]. GA3 application synthesis restores the photosynthetic efficiency [42], improves growth [26], and decreases oxidative damage [53,99]. External kinetin supplementation enhanced the proline content in different plants under abiotic stress, viz., in *Vigna angularis* under cadmium stress, in *Salvia officinalis* under salt stress [100], and in tomato under salt stress [101]. Kinetin-induced proline accumulation may be attributed to the decline in activity of proline dehydrogenase [20], improved ROS scavenging, and prevention of photoinhibition [102].

Cr-stress-enhanced ROS production has been reported by several works [4,103]. Under stressful conditions, H_2_O_2_ plays a central role in signaling [104,105]. However, it is also intended to be the key ROS molecule that negatively affects plant growth and development. The Cr stress induces lipid peroxidation and increases the production of MDA [37,106], which, in turn, disturbs the fluidity and integrity of biological membranes [107,108], and the H_2_O_2_ also obstructs the Calvin cycle, causing disruption in the rates of photosynthesis [109,110]. However, our study revealed that the application of kinetin and GA3 reduced the H_2_O_2,_ MDA, and EL in Cr-stressed faba bean plants. Kinetin is known to shield the cell membrane components by recovering the plants from lipid peroxidation induced by heavy metal stress. Kinetin also causes a significant reduction in H_2_O_2_ and MDA content by increasing antioxidant activities that help in quenching the ROS [19,101]. Additionally, the GA3 application also reduced ROS accumulation under salt stress [111], cadmium stress [23] and boron stress. Kinetin and GA3 application in faba bean plants mitigate Cr stress by enhancing antioxidant activities, which, in turn, scavenge ROS molecules and prevent membrane damage.

Metal toxicity in plants inevitably results in oxidative stress due to ROS over-production [54,58]. Plants have developed an intricate and robust antioxidant defense system based on enzymatic and nonenzymatic antioxidants to counteract such situations [112,113]. In the present study, we observed an upsurge in both enzymatic and nonenzymatic antioxidants under Cr toxicity. Our results corroborate the observations in *Helianthus annuus* [98] and tomato [38]. However, the application of GA3 and kinetin moderated the enzymes of the AsA-GSH cycle differently, by changing APX, MDHAR, and DHAR activities to a varying level. Moreover, kinetin treatment prompted an enhancement in GR activity. A well-mediated redox status is likely sustained by the application of kinetin and GA3 that may help to renew GSH from GSSG, which is clearly evident from the increase observed in the levels of GSH together with GSG/GSSG ratio [18]. Plants under different environmental perturbations can activate the GSH-dependent defense mechanisms that play an important role in plant protection. The defense mechanism associated with AsA-GSH under the supplementation of kinetin and GA3 established further improved GSH levels [101]. This might be the reason behind the biosynthesis and modulation of GR activity under Cr stress, which might have subsequently contributed in the GST and GPX-facilitated effectual decontamination of hydroperoxides. Our results are in agreement with those observed in *Pisum sativum* under chromium stress [114] and *Vigna angularis* under cadmium stress, and they demonstrated an increase in the AsA-GSH cycle enzymes. Similar findings were also reported by Hassan and Mansoor [115] in mung bean. In addition to kinetin, several authors have also demonstrated a positive role of GA3 in stress tolerance [23,53,99]. Hence, the application of kinetin and GA3 alone or combination might prevent the plant cells from oxidative damage by maintaining osmotic balance, preventing Cr translocation and enhancing antioxidative enzymatic machinery, and maintaining the proper redox balance.

It has been reported that in plants, the GSH-dependent Gly system is associated with the maintenance of the redox balance and detoxification of MG [116]. The current study was carried out with the aim to decipher the role of kinetin and GA3 application in improving Cr stress tolerance. During the current study, it was found that the MG concentration was enhanced under Cr stress likely because of the inefficient working of the glyoxylase enzyme system due to toxicity caused by Cr. The application of kinetin and GA3 enhanced GlyI and GlyII activities that might have helped the plants to overcome the cytotoxic effects of MG. Several studies have deliberated about the increased levels of glyoxylase enzymes under stressful conditions [117].

In conclusion, the present study is the first report demonstrating the combined effect of kinetin and GA3 in the Cr tolerance in faba bean. Our results revealed that the combined application of kinetin and GA3 has more positive effects on the accumulation of osmolytes, antioxidant enzyme activities, glyoxylase enzyme activities, and efficient ROS detoxification. Hence, our results suggested defense crosstalk between kinetin and GA3 in the regulation of Cr stress tolerance in faba bean. However, the mechanism underlying this crosstalk needs to be explored in the future to harness the true potential of these phytohormones in the regulation of Cr tolerance in faba bean.

## 4. Materials and Methods

### 4.1. Plant Growth and Treatments

The seeds of faba bean (*Vicia faba* L.) were disinfected by using 70% ethanol for 8 min, and the disinfected seeds were further surface-sterilized with 4% sodium hypochlorite (NaOCl) for 15 min by following the procedure of Alsahli et al. (2020). After surface sterilization, the seeds were thoroughly rinsed with double-distilled water to remove the surface chemicals. After sterilization, the seeds were primed with 10 µM kinetin solution for 12 h, and the priming was performed by dissolving kinetin in 1 N NaOH and then diluting to 10 µM concentration by adding deionized water [118]. For the control treatment, the seeds were just placed simply in deionized water for 12 hrs. Following the kinetin treatment, the seeds were air-dried and subsequently sown in the earthen pots (10 seeds/pot) containing soil, sand, and vermiculite in the ratio of 1:1:1 (Table 2). After germination, three seedlings were maintained in each earthen pot in a completely randomized-block-design (RBD) manner, and each replicate included five plants. The 11-day-old seedlings were subjected to Cr stress (300 μM) by dissolving potassium dichromate (K_2_Cr_2_O_7_) in nutrient solution. Control plants were provided with the nutrient solution only. The Cr was given every week (two days per week, 50 mL per pot) until the end of the experiment (31 day). Gibberellic acid (GA3) at the rate of 10^−6^ M mixed with Tween-20 was applied to the leaf tissues after every alternate day from the first day of Cr treatment. The faba bean plants were raised in a light chamber under controlled conditions with day/night temperatures of 28/22 °C, photosynthetically active radiation (PAR 200 μmol m^−2^ s^−1^), and a relative humidity of 65%. The collection of leaf samples was carried out after two weeks of the treatment to perform different biochemical and physiological analysis. Overall, our experiment consisted of the following seven treatments:

- 0 mM (Control);
- 0 mM + Kinetin (10 µM);
- 0 mM + GA3 (10^−6^ M);
- 0 mM + Kinetin (10 µM) + GA3 (10^−6^ M);
- Cr (300 μM);
- Cr (300 μM) + Kinetin (10 µM);
- Cr (300 μM) + GA3 (10^−6^ M);
- Cr (300 μM) + Kinetin (10 µM) + GA3 (10^−6^ M).

**Table 2:** Analysis of media used in the experiment.

Parameters	
Texture	Sandy loam
pH	8.83
Cr	ND
Organic matter	1.12

ND: not detected.

### 4.2. Plant Growth Parameters

Shoot and root length measurements of different faba bean plants were carried out by using a manual scale. For dry weight estimation, the plant samples were oven-dried at 70 °C for 48 h, and the dried material was then weighed using a weighing balance. We followed the procedure of Alsahli, et al. [119] to measure these parameters.

### 4.3. Estimation of Chromium

For the Cr estimation, a sample of 0.5 g of dried plant material was acid-digested in HNO_3_ (70%) using the microwave digestion system 2000, following the procedure as described by Gupta and Sinha [120]. In dried leaf samples, the Cr content was estimated with the help of an atomic-absorption spectrophotometer (Perkin Elmer AA700, Waltham, MA, USA). The Cr accumulation and translocation were estimated as per Duman et al. [121].

### 4.4. Pigment Content, Photosynthesis, and Gas Exchange Parameters

Measurements pertaining to chlorophyll were carried out by following the method of Lichtenthaler and Wellburn [122]. The 0.5 g fresh leaf sample was homogenized with 5 mL of acetone (80% *v*/*v*) and centrifuged at 10,000× *g* for 8 min. The absorbance of samples was recorded at 480, 510, 645, and 663 nm by using the spectrophotometer (Beckman 640D, Brea, CA, USA). A Pulse Modulation Fluorometer (PAM 2500; Heinz Walz GmbH, Pfullingen, Germany) was used to perform measurements related to chlorophyll fluorescence [123]. Photosynthetic parameters including Pn (net photosynthetic rate), A (Carbon dioxide assimilation), *gs* (stomatal conductance), and *E* (Transpiration rate) were assayed (9:00–11:00 am) by operating a portable photosynthesis system IRGA (LI-COR, Lincoln, NE, USA).

### 4.5. Analysis of LRWC and Proline and Glycine Betaine Content

Leaf relative water content (RWC) was assayed by the method as previously demonstrated by Barrs and Weatherley [124]. The LRWC was estimated by using the formula of Avestan, et al. [125]

(1) $$\mathrm{LRWC}=\frac{\mathrm{FW}-\mathrm{DW}}{\mathrm{TW}-\mathrm{DW}}\times 100$$

For the estimation of proline, the method of Bates, et al. [126] was used. The optical density of the supernatant was then measured at 520 nm by using a spectrophotometer. Toluene was used as the blank. Glycine betaine (GB) was assayed as per the method of Grieve and Grattan [127].

### 4.6. Estimation of Hydrogen Peroxide (H_2_O_2_), Lipid Peroxidation (Malondialdehyde), and Electrolyte Leakage

Hydrogen peroxide levels were assayed as described previously by Velikova, et al. [128]. Lipid peroxidation measurement, expressed as thiobarbituric acid reactive substances (TBARS) content, was carried out following the method of Heath and Packer [129].

To determine leaf relative electrolyte leakage (LREL), the fully expanded upper fourth leaves from faba bean plants were excised and rinsed with deionized distilled water. Ten leaf discs (5 mm in diameter) punched from the leaf material were placed in glass bottles containing 15 mL of distilled deionized water and then shaken for 4 h at 300× *g* in the dark at 25 °C. With the help of a conductivity detector (DDS SJ-308A, Shanghai, China), the electrolyte leakage (R1) was measured in the solution at 25 °C. Then, after the solution containing leaf discs was allowed to boil for 20 min and subsequently cool down to room temperature, electrolyte leakage (R2) was measured from the boiled solution at 25 °C. LREL (%) was calculated as:

(2) Electrolyte leakage%=EC1/EC2×100

### 4.7. Enzyme Extraction and Assays

Enzymes were extracted from 0.1 gm of 21-day-old frozen leaf samples with 1 mL of freshly prepared extraction buffer containing (50 mM potassium phosphate buffer pH 7.8, 2 mM Na_2_-EDTA, 10 mM 1,4, dithiothreitol, 20 mM ascorbic acid, 0.6% PVPP, and 50 µL of protease inhibitor cocktail). The extracted samples were centrifuged for 15 min at 14,000× *g* at 4 °C to obtain the supernatant for enzyme assays. The enzyme activity was expressed as EU mg^−1^ of protein.

Total superoxide dismutase activity (SOD; 1.15.1.1) was assayed according to Dhindsa and Matowe [130]. The protocol of Aebi [131] was employed for the estimation of catalase activity (CAT;1.11.1.6). Ascorbate activity (APX;1.11.1.11) was measured by monitoring the decrease in absorbance at 290 nm for 3 min from the enzyme extract containing H_2_O_2_ and ascorbic acid (Nakano and Asada [132]). Glutathione reductase (GR;1.6.4.2) activity was assayed by the method of Foster and Hess [133]. Monodehydroascorbate reductase (MDHAR; 1.6.5.4) activity from the extract was assayed as per Miyake and Asada [134]. Dehydroascorbate reductase (DHAR; 1.8.5.1) was assayed as per the method of Nakano and Asada [132]. GST (EC 2.5.1.18) activity was measured following the method of Alsahli et al. [119]. Protein contents in the enzyme extracts were determined using Coomassie brilliant blue G-250 [135].

### 4.8. Nonenzymatic Antioxidants

The method of Huang, et al. [136] was followed to access the levels of ascorbate (AsA) and the protocol by Yu, et al. [137] was used for the estimation of glutathione pool.

### 4.9. MG Content Estimation

MG content was assayed by the method previously described by Wild, et al. [138]. Glyoxalase I and glyoxylase II were estimated by the method described by Alsahli et al. [119].

### 4.10. Statistical Analysis

One-way analysis of variance (ANOVA) and Tukey’s HSD (Honestly Significant Difference) tests were used to achieve the statistical analysis with a significance level of 0.05 on all results using SPSS v.17 software.

## 5. Conclusions

The stress induced by Cr was effectively mitigated by kinetin along with GA3 as photosynthetic efficiency was strengthened, oxidative damage was reduced, and growth attributes were recovered. In addition to these functions, the application of kinetin and GA3 together showed excellent efficacy in boosting osmolyte accumulation and efficiently detoxifying ROS by enhancing the capacity of both enzymatic and nonenzymatic antioxidants to prevent oxidative damage. Furthermore, by regulating the GSH-based Gly systems to detoxify MG, the combination of kinetin and GA3 application played a critical function in maintaining the AsA/DHA and GSH/GSSG ratios that also assisted in reducing oxidative damage. The study suggests that kinetin along with GA3 are effective protectants that improve Cr tolerance in faba bean plants by enhancing antioxidant enzymes and reducing oxidative damage. Nevertheless, under Cr stress, kinetin and GA3-mediated resilience in plants and its mechanism should be deeply investigated at the molecular level.

## Figures and Tables

**Table 1:** Effect of KN and GA3 on Cr accumulation by shoot and root and translocation factor in faba bean under Cr toxicity. The letters (a–d) denote significant difference at *p* < 0.05. Data represent mean ± SE (n = 5).

Treatments	Shoot Cr (mg kg^−1^ DW)	Root Cr (mg kg^−1^ DW)	Translocation Factor (TF)
0	ND	ND	ND
0 + KN (10 µM)	ND	ND	ND
0 + GA3	ND	ND	ND
KN + GA3	ND	ND	ND
Cr (300 µM)	31.43 ± 0.727 a	57.48 ± 1.32 a	0.546 ± 0.014 a
Cr + Kn	19.25 ± 0.424 c	42.15 ± 0.97 b	0.456 ± 0.008 c
Cr + GA3	22.71 ± 0.565 b	45.67 ± 1.13 b	0.497 ± 0.011 b
Cr + Kn + GA3	15.48 ± 0.311 d	32.72 ± 0.65 c	0.473 ± 0.008 bc

ND: Not detected.

## Data Availability

The data are available in the manuscript.
